# Behavioral and neurophysiological effects of an intensified robot-assisted therapy in subacute stroke: a case control study

**DOI:** 10.1186/s12984-020-00792-1

**Published:** 2021-01-11

**Authors:** Aida Sehle, Jana Stuerner, Thomas Hassa, Stefan Spiteri, Mircea A. Schoenfeld, Joachim Liepert

**Affiliations:** 1 Lurija Institute and Department of Neurological Rehabilitation, Kliniken Schmieder, Zum Tafelholz 8, 78476 Allensbach, Germany; 2 Department of Neurological Rehabilitation, Kliniken Schmieder, Heidelberg, Germany; 3 Department of Neurology, Otto-Von-Guericke-University Magdeburg, Magdeburg, Germany; 4 Leibniz Institute for Neurobiology, Magdeburg, Germany

**Keywords:** Stroke, Robotics, Upper extremity, Dose–response relationship, Motor recovery, Transcranial magnetic stimulation

## Abstract

**Background:**

Physical training is able to induce changes at neurophysiological and behavioral level associated with performance changes for the trained movements. The current study explores the effects of an additional intense robot-assisted upper extremity training on functional outcome and motor excitability in subacute stroke patients.

**Methods:**

Thirty moderately to severely affected patients < 3 months after stroke received a conventional inpatient rehabilitation. Based on a case–control principle 15 patients were assigned to receive additional 45 min of robot-assisted therapy (Armeo^®^Spring) 5 times per week (n = 15, intervention group, IG). The Fugl-Meyer Assessment for the Upper Extremity (FMA-UE) was chosen as primary outcome parameter. Patients were tested before and after a 3-week treatment period as well as after a follow-up period of 2 weeks. Using transcranial magnetic stimulation motor evoked potentials (MEPs) and cortical silent periods were recorded from the deltoid muscle on both sides before and after the intervention period to study effects at neurophysiological level. Statistical analysis was performed with non-parametric tests. Correlation analysis was done with Spearman´s rank correlation co-efficient.

**Results:**

Both groups showed a significant improvement in FMA-UE from pre to post (IG: + 10.6 points, control group (CG): + 7.3 points) and from post to follow-up (IG: + 3.9 points, CG: + 3.3 points) without a significant difference between them. However, at neurophysiological level post-intervention MEP amplitudes were significantly larger in the IG but not in the CG. The observed MEP amplitudes changes were positively correlated with FMA-UE changes and with the total amount of robot-assisted therapy*.*

**Conclusion:**

The additional robot-assisted therapy induced stronger excitability increases in the intervention group. However, this effect did not transduce to motor performance improvements at behavioral level.

*Trial registration* The trial was registered in German Clinical Trials Register. Clinical trial registration number: DRKS00015083. Registration date: September 4th, 2018. https://www.drks.de/drks_web/navigate.do?navigationId=trial.HTML&TRIAL_ID=DRKS00015083. Registration was done retrospectively

## Introduction

Stroke patients frequently suffer from motor deficits [1]. The prognosis in severely affected individuals is poor with about 60% failing to achieve at least some dexterity at 6 months after stroke [2]. Thus, further reduction of these deficits is a major challenge for rehabilitation. Various techniques as constraint-induced movement therapy, mirror therapy, virtual reality, neuro-muscular electrical stimulations, task-oriented training and the use of (electro)-mechanical devices to support motor rehabilitation have been recommended so far [3, 4]. Lately, electro-mechanical and robot-driven devices were shown to be effective regarding activities of daily living as well as arm and hand function [5]. However, the authors pointed out that intensity, duration, amount and type of training, device type, participants´ characteristics varied in studies included in their meta-analysis leading to lower evidence quality [5, 6]. Other authors also emphasized that detailed recommendations regarding training intensity and frequency are missing [7]. However, some evidence is available indicating that more movement practice leads to better outcomes [8, 9]. Furthermore, it was recommended to increase exercise intensity by making the tasks more difficult and/or increasing the number of repetitions [10, 11]. Presumably, robot-assisted therapy is effective because it allows to deliver both: high-dosage and high-intensity training [12].

Most studies that evaluated an intense training program have been conducted in chronic stroke patients [13–18]. Much less is known about the impact of robot-assisted therapy as well as the dosage for the paretic upper limb within the first 3 months after the stroke [7].

Moreover, the results of rehabilitation are usually presented as improvements in motor function using behavioral measures. Our understanding of the pathophysiology of motor dysfunction and recovery is still limited [19]. In order to optimally design the rehabilitation program, a more complete understanding of the physiological processes of recovery is required [19]. Transcranial magnetic stimulation (TMS) as a suitable tool for safe and painless examination of cortical and corticospinal physiology could help to clarify these processes [20]. Previous studies in stroke patients have shown an enlargement of motor cortex representations after exercises as well as due to spontaneous functional recovery [21–23].

To address some of the raised topics, we investigated the effects of an additional robot-assisted training of the upper extremity in subacute stroke patients. For the training we used an upper extremity exoskeleton that provides an adjustable arm support and allows gravity-supported and computer-enhanced arm exercises (Armeo^®^Spring). Several studies using this device have already demonstrated improvements in motor functions, including increases of strength, as well as reductions of spasticity and pain [24–26].

The present study was performed in patients who received a multidisciplinary inpatient rehabilitation. It was designed to answer three questions:

1. Does this intensified treatment lead to clinical improvements?
2. Does the additional training induce motor excitability changes and how do these relate to improvements of motor functions?
3. Is it feasible to increase the amount of motor training relatively early after a severe stroke or would patients discontinue their participation, e.g. due to too much fatigue?

## Methods

### Trial design

**Fig. 1:**
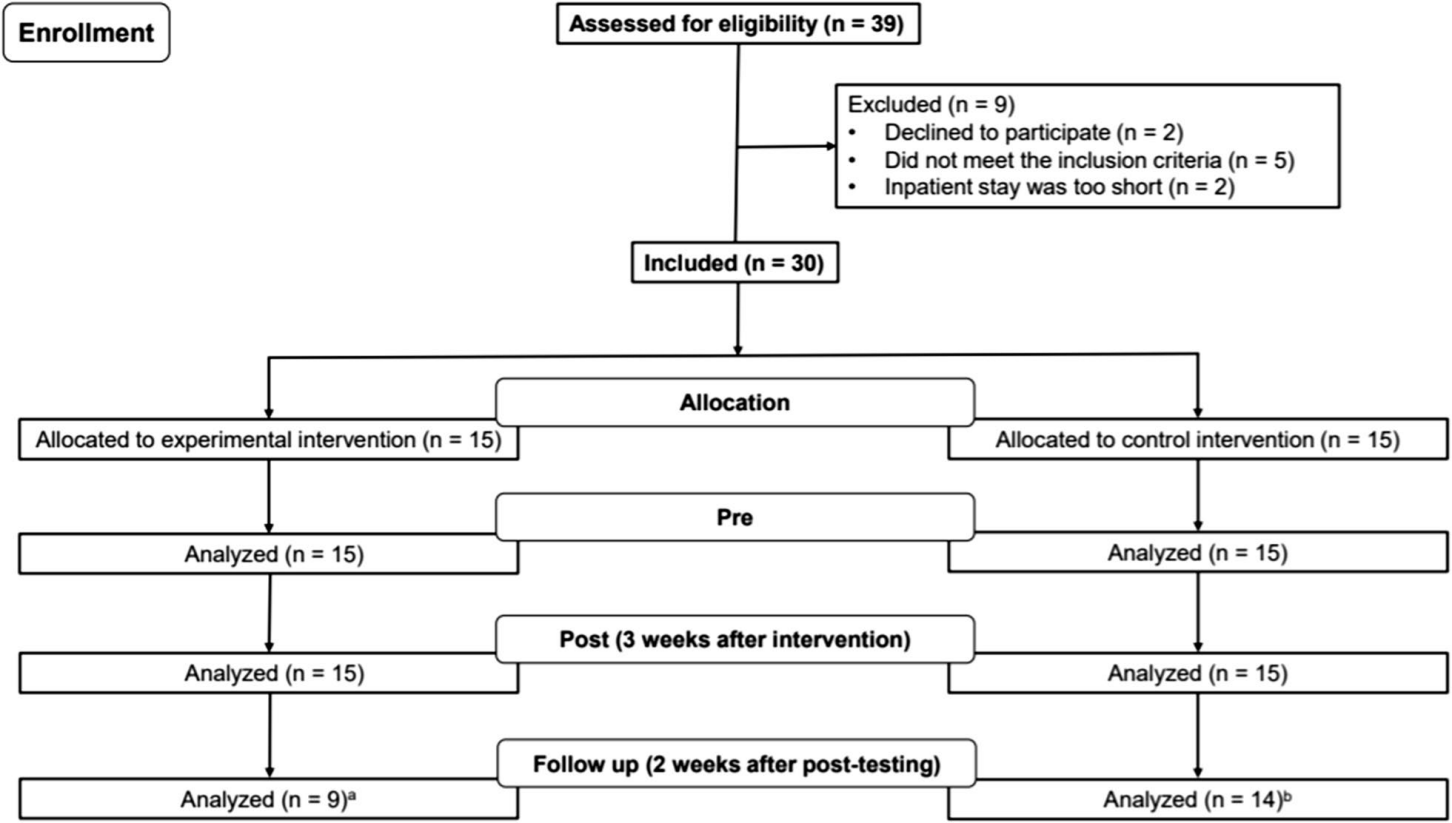
Chart flow of patient recruitment and participation

The study design was a prospective, single-blind case control study in subacute poststroke patients. Figure 1 shows the selection process in the study.

The primary outcome parameter was a change in upper extremity function measured by the Fugl-Meyer Assessment for the upper extremity (FMA-UE) [27]. As secondary parameters TMS criteria (size of the motor evoked potential and duration of the cortical silent period obtained during voluntary muscle activation [“pre-innervation”]) were chosen. The investigators who administered and scored the behavioral tasks and the neurophysiological experiments were blinded for the type of training the patients received.

The study protocol and informed consent form were approved by the local ethics committee of the University of Konstanz, Germany. The study was conducted in accordance with the Declaration of Helsinki. All participants were informed regarding the experimental nature of the study. Written informed consent was obtained from all subjects. The trial was registered in German Clinical Trials Register. Clinical trial registration number: DRKS00015083.

### Subjects

A total of 30 subacute stroke patients with severe or moderate-to-severe upper-limb hemiparesis were included in the study (Table 1). Patients were recruited from the department of Neurorehabilitation (Kliniken Schmieder, Allensbach, Germany). Patients were assigned to the intervention or control group according to the case control principle. The baseline FMA-UE values and the patients´ age were used as matching criteria. Each group consisted of 15 participants.

**Table 1:** Demographic and clinical data

	Gender (m/f)	Age (years)^a^	Time since incident (weeks)^a^	Lesion location (subcortical^b^/cortico-subcortical)	FMA-UE baseline (total score)^a^	MoCA^a^	Severe limb apraxia	Severe neglect
Intervention group (n = 15)	8/7	61.4 ± 14.3	7.1 ± 3.5	8/7	20.7 ± 11.6	21.9 ± 5.6	no	no
Control group (n = 15)	6/9	60.7 ± 11.9	8.1 ± 5.1	8/7	18.7 ± 9.2	21.3 ± 4.9	no	no

m, male; f, female, FMA-UE, Fugl-Meyer Assessment for Upper Extremity; MoCa, Montreal Cognitive Assessment

^a^Values are presented as mean ± 1 standard deviation

^b^Subcortical without cortical involvement

The inclusion criteria for this study were: 1. less than 3 months after first stroke (ischemia or hemorrhage); 2. presence of a severe or moderate-to-severe upper limb paresis; 3. at least 18 years of age; 4. sufficient understanding of instructions; 5. ability to give informed consent for the participation in the study; 6. ability to operate the Armeo^®^Spring; 7. movement of the arm possible if the weight of the arm is carried by a support (at least strength level 2 for shoulder joint movement).

The exclusion criteria were: 1. prior history of neurological illness or psychiatric conditions; 2. cognitive impairments that interfere with understanding the instructions (e.g. receptive aphasia, global aphasia, and dementia); 3. A score of less than 26 points in the Montreal Cognitive Assessment (MoCA) [28]; 4. participants demonstrating insufficient compliance; 5. severe apraxia; 6. severe pain associated with movements of the affected upper extremity (> 3 on the visual analogue scale [scale ranging from 0 to 10]).

### Experimental procedure

Robotic-assisted therapy was conducted using the Armeo^®^Spring exoskeleton (Hocoma AG, Zurich, Switzerland) which provides a weight support for the upper extremity and is connected to a PC with which various virtual-reality based games can be played. All patients participated in a conventional inpatient neurorehabilitation. Importantly during inpatient rehabilitation both groups received low-dose Armeo^®^Spring treatment (two times a week × 30 min) as a therapeutic intervention within the standard rehabilitation procedures at the clinic. The intervention group received an additional 45 min training session with the Armeo^®^Spring five times a week for three weeks. The conventional Armeo^®^Spring treatments were conducted in the afternoon, the additional training sessions took place in the morning.

### Behavioral measures

#### Fugl-Meyer assessment for upper extremity (FMA-UE)

In order to record changes of motor function, the patients were tested with the FMA-UE at baseline, after three weeks (post) and after another two weeks of inpatient rehabilitation (follow-up) [27]. The follow up interval of two weeks (instead of three weeks or an even longer period) was chosen in order to maximize the probability that the patient was still available as an inpatient for testing. The FMA-UE is a widely used quantitative measure of motor recovery post stroke (score range, 0–66; with higher scores indicating better performance) [27]. Functional improvements of five points or more are considered to indicate a clinically meaningful change [4, 29].

### Assessment of limb apraxia

Standardized tests were used to assess limb apraxia in all patients at baseline [30, 31]. The performance in imitating meaningful as well as meaningless hand-gestures (each max = 20 points) and the pantomimic use of familiar objects (max = 16 points) was evaluated. Detailed assessment procedures, psychometric data and cut-off values were derived from Randerath et al. [32].

### Assessment of visuospatial neglect (Bells test)

Bells test was applied at baseline for evaluation of visuospatial neglect. It is one of the most widely used instruments for the diagnosis of visuospatial neglect developed by Gauthier et al. [33]. The task consists of an array of bells and unrelated distractors, and permits a quantitative evaluation of visuospatial neglect [33, 34]. The maximum score is 35. An omission of six or more bells in the contralateral visual field argues for the presence of visuospatial neglect [34].

### Neurophysiological measures

#### Transcranial magnetic stimulation (TMS)

In 25 subjects, TMS was performed over the motor cortex using a round coil that was attached to a Magstim 200 device (The Magstim Company Ltd, United Kingdom) at baseline and after 3 weeks. The other five subjects were excluded due to TMS contraindications.

Motor evoked potentials (MEPs) and cortical silent periods (cSP) were recorded from both deltoid muscles consecutively. The reason for choosing the deltoid muscle as the target was the expectation to record MEPS and cSPs more frequently from this muscle than from a hand muscle. First, the best coil position was defined as the position where MEPs were evoked with the lowest stimulus intensity. This position was marked with ink on the skull of the patient. The motor threshold (MT) was then determined by adjusting the stimulus intensity. MT was defined as an intensity that produces MEPs of > 50 µV in at least 5 of 10 trials [35]. Data analysis was performed offline.

### MEP amplitudes at rest

Single TMS pulses were used to test the corticomotor excitability. These were applied with 120% motor threshold intensity. Both hemispheres were tested consecutively. Five stimuli were given and a mean value was calculated. Recordings were obtained while the subject was at rest.

### MEP amplitudes during pre-innervation and cortical Silent Period (cSP)

Transcranial magnetic stimulation was applied while the subject performed an isometric contraction of the deltoid muscle at about 20% of maximum voluntary contraction (MVC). First, the unaffected hemisphere was stimulated, followed by stimulation of the affected hemisphere. Five stimuli were given consecutively. The cSP duration was analyzed by measuring the time from MEP onset until the re-occurrence of EMG activity. MEP amplitudes were measured peak-to-peak. Mean values were calculated for both parameters. MEP amplitudes were used as an indicator of excitation whereas the cSP reflects inhibitory properties [36, 37].

### Feasibility

The main parameter to determine the feasibility was the dropout rate. When patients stopped participating in the study, it was important to find out why (e.g. due to fatigue, pain or difficulty to operate the device). It was also recorded if adverse events occurred during the study.

### Statistics

The statistical analysis of the behavioral and TMS data were conducted using IBM SPSS Statistics 25. Only patients with complete datasets were analyzed. The data was first tested for normal distribution using the Shapiro–Wilk test which showed that the data was not normally distributed (p < 0.02). Consequently, non-parametric tests were used for further analysis. Friedman’s test was used to compare the mean outcome at multiple time points (repeated measurements). The Wilcoxon test was applied to calculate differences between two time points. Between group comparisons were calculated using the Mann–Whitney-U test. Correlations were calculated using Spearman’s correlation *r*_*s*_. The level of significance for all tests was set at α = 0.05.

## Results

### Behavioral measures

All included patients neither showed apraxic behavior, nor visuospatial neglect and were cognitively capable of understanding the instructions and executing the tasks.

**Fig. 2:**
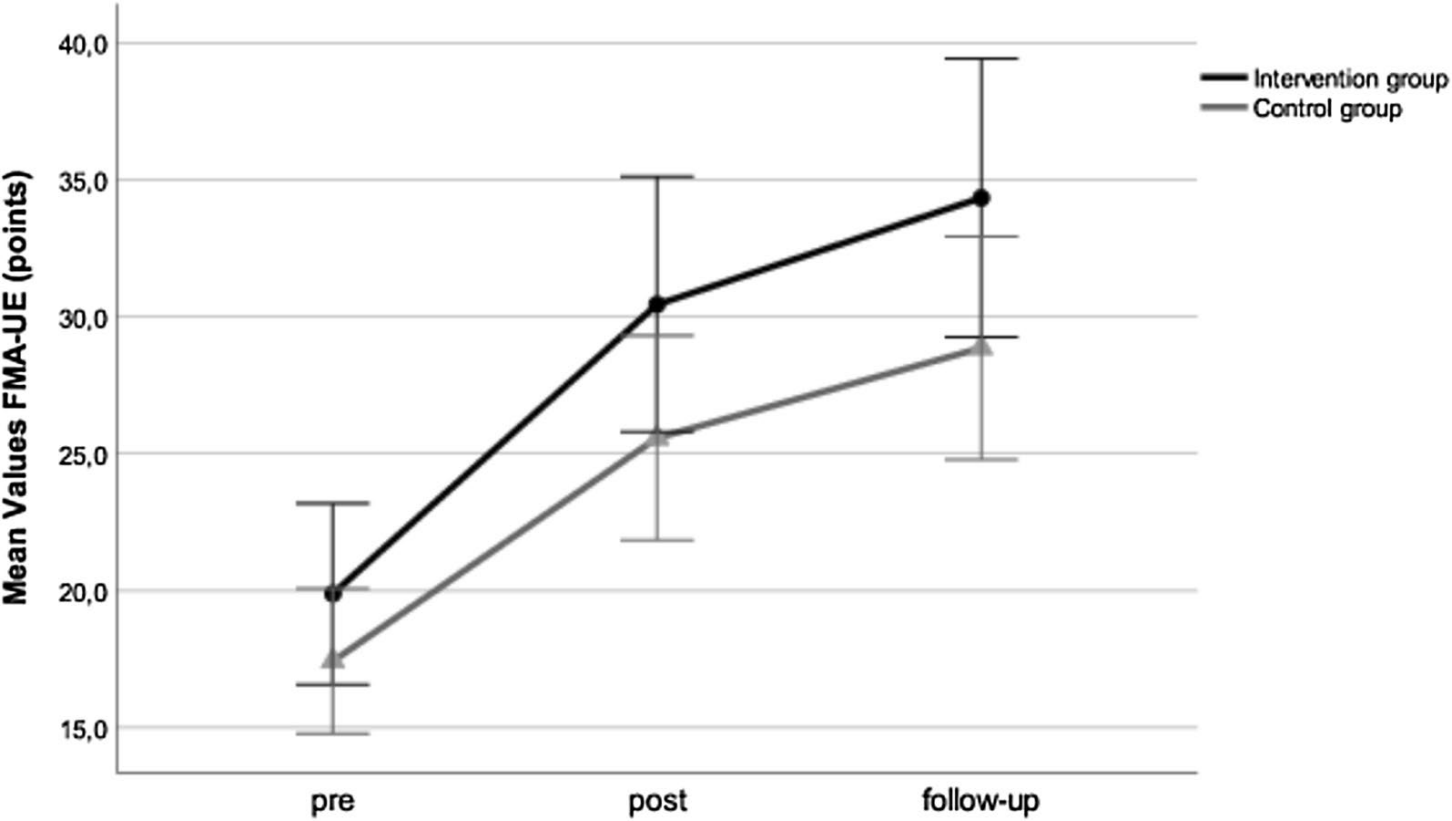
Changes of the Fugl-Meyer Assessment for Upper Extremity (FMA-UE) for both groups and three measurement times. Mean values and ± 1 standard error are displayed

In the intervention group, the mean FMA-UE score improved by 10.6 points from baseline to post-testing and by 3.9 points from post-testing to follow-up. In the control group, the mean FMA-UE score improved by 7.3 points from baseline to post-testing and by 3.3 points from post-testing to follow-up. Both groups improved significantly in the FMA-UE (p < 0.001) (Fig. 2). Furthermore, the data analysis showed significant functional improvements between all three measurement times in mean FMA-UE (p < 0.04) for each group. However, no significant differences were observed between groups at any of the three time points (p > 0.30).

It is however noticeable, that none of the patients (n = 4) with baseline values ≤ 10 points on FMA-UE showed clinically meaningful changes of five points and more. Ten of 13 patients with FMA-UE values between > 10 und ≤ 20 points reached clinically meaningful improvements. Three of four patients with FMA-UE values between > 20 und ≤ 30 points showed a change of 5 points or more. All patients with FMA-UE values above 30 points on FMA-UE (n = 6) achieved clinically meaningful changes. Three patients belonging to the control group developed shoulder pain during the treatment phase. Thus, they worsened in the post test.

The correlation analysis showed no significant correlation between the baseline FMA-UE and the degree of functional improvement (*r*_*s*_ = 0.22; p = 0.25). Furthermore, there was no significant correlation between the amount of upper extremity-focused therapy and the degree of functional improvement (*r*_*s*_ = 0.23; p = 0.23).

### Neurophysiological measures

Table 2 shows neurophysiological parameters. This table only includes complete datasets.

**Table 2:** Mean and standard deviation of neurophysiological measures

TMS variables	Paretic side^a^		Non-paretic side^a^	
	*Pre*	*Post*	*Pre*	*post*
MEPs during pre-innervation *(mV)*				
Intervention group	1.95 ± 2.7 (n = 10)	3.3 ± 3.02 (n = 10)	5.3 ± 2.1 (n = 12)	5.1 ± 2.2 (n = 12)
Control group	1.1 ± 0.7 (n = 13)	1.2 ± 0.6 (n = 13)	5.2 ± 2.7 (n = 13)	5.9 ± 3.3 (n = 13)
Cortical silent period *(ms)*				
Intervention group	147.0 ± 40.7 (n = 6)	134.7 ± 18.2 (n = 6)	69.7 ± 14.0 (n = 12)	68.8 ± 14.0 (n = 12)
Control group	165.4 ± 49.3 (n = 7)	160.5 ± 48.5 (n = 7)	82.8 ± 25.2 (n = 13)	83.0 ± 24.4 (n = 13)

TMS, transcranial magnetic stimulation; MEPs, motor evoked potentials; mV, millivolt; ms, millisecond

^a^Values are presented as mean ± 1 standard deviation

The neurophysiological parameters on the non-paretic side remained stable and did not differ significantly for both pre-post and between group comparisons (p > 0.05). Furthermore, the neurophysiological parameters differed significantly at baseline between the affected and healthy sides in both groups (p < 0.002).

#### MEP amplitudes at rest on the paretic side

In 21 patients the resting motor threshold in the affected hemisphere exceeded 100% of stimulator output intensity. Therefore, MEP responses could only be derived from the relaxed deltoid muscle in four of the 25 patients. After three weeks, MEP responses could be derived from three additional patients. Due to the small number of cases no further statistical evaluation was done.

#### MEP amplitudes during pre-innervation on the paretic side

At baseline, MEPs could be obtained on the paretic side in all except for two patients. After three weeks, one of these two patients showed a MEP during pre-innervation. In the intervention group, a significant increase in the MEP amplitudes in the post-test compared to the pre-test was found on the paretic side (p < 0.01). In the control group, no difference in the MEP amplitudes in the post-test compared to the pre-test was found (p = 0.6). In addition, there was no difference between the groups at baseline (p = 0.44) but in post-testing (p = 0.02). The MEP amplitudes of the intervention group were significantly higher than in the control group.

#### Cortical Silent Period on the paretic side

At baseline, cSP could be clearly determined on the paretic side in 13 of the 25 patients. In the other patients the cSP was not clearly definable. After three weeks, the cSP could be determined in three additional patients. The duration of cSP neither showed differences in the pre-post, nor in between groups comparisons (p > 0.24).

#### Correlation analysis

**Fig. 3:**
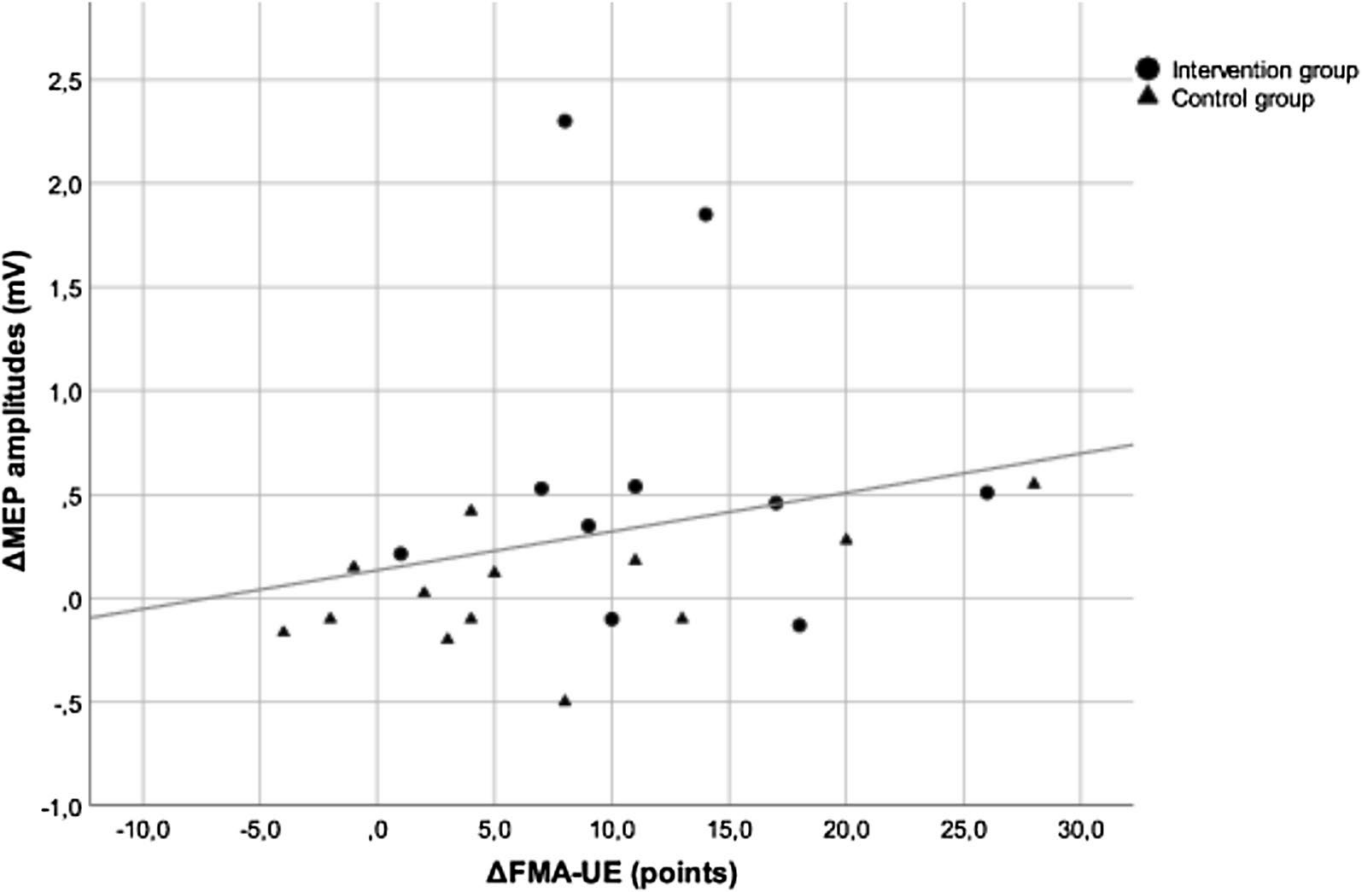
Correlation between the changes of MEP amplitudes during pre-innervation on the paretic side and the degree of clinical improvement. MEP, motor evoked potentials; mV, millivolt; FMA-UE, Fugl-Meyer Assessment for Upper Extremity

On the paretic side, changes of MEP amplitudes showed a positive correlation with the degree of clinical improvement (*r*_*s*_ = 0.43; p < 0.04) (Fig. 3). Similarly, changes of MEP amplitudes during pre-innervation on the paretic side showed a positive correlation with the amount of upper extremity-focused therapy (*r*_*s*_ = 0.49; p < 0.02) (Fig. 3). However, cSP changes neither correlated with the degree of clinical improvement nor with the amount of therapy (*r*_*s*_ = − 0.41; p = 0.16 and *r*_*s*_ = − 0.08; p = 0.79).

### Feasibility

None of the patients stopped participating in the study prematurely. The patients of the intervention group sometimes found the additional appointments challenging but feasible. Three patients belonging to the control group developed shoulder pain during the treatment phase. These patients showed some deterioration in the post-treatment FMA-UE. None of the patients in the intervention group developed shoulder pain.

## Discussion

The main result of this study, based on the FMA-UE assessment, indicates that both patient groups improved to the same extend. Thus, a positive effect of the high-dose treatment with the Armeo^®^Spring could not be observed at behavioral level. Several reasons may account for this result. First, within the first months after stroke, spontaneous recovery still occurs. According to the proportional recovery rule, the degree of functional improvement within the first months after stroke can be predicted in patients with mild to moderate initial impairments [38, 39]. However, our patient group rather belongs to the subgroup of patients for whom a prediction is much less reliable. Earlier studies have demonstrated that, in this patient group, some subjects show recovery and others do not [38, 39]. Typically, the strongest improvements are observed within the first 12 weeks poststroke [40–43]. Thus, improvements evoked by spontaneous recovery can be expected in both patient groups to a similar degree, since the mean time since stroke onset was almost identical. Spontaneous recovery might also have reduced the effect size of the intervention.

Second, the additional amount of therapy might not have been sufficient to induce a superior clinical improvement. Indirect evidence gained from studies with patients in the chronic phase after stroke suggests that 15–30 additional hours of therapy did not make a difference [15, 44, 45]. However, clinical studies that provided 90–300 h of therapy could demonstrate a significant improvement [14, 17]. Thus, the additional amount of therapy possibly needs be much higher than in our study to make a difference at behavioral level. However, results obtained in chronic stroke patients may not be the same in subacute patients. In terms of feasibility, it is quite difficult to deliver training with such an extreme intensity within the framework of an inpatient rehabilitation. In our patients, treatment was not restricted to upper limb therapies but patients also had treatments for trunk control, walking and balance, and a majority also received language and swallowing therapies. Thus, the daily schedule was filled with a large variety of therapeutic approaches aiming at different therapeutic targets. The implementation of an even higher amount of therapies is most difficult also because of patient-associated limitations, e.g. fatigue.

Third, both patient groups received approximately 5 h of upper limb therapies per week as conventional inpatient treatment program. Presumably, conventional treatments had a relevant influence on the patients’ improvement. This might have masked or even diminished beneficial effects of the Armeo^®^Spring intervention.

Compared to other studies, patients in this study had a clinically meaningful increase in performance in both groups above 5 FMA-UE points [7, 46].

In our study, patients with an initial FMA-UE [27] below 10 points showed no clinically relevant improvements. Unfortunately, the limited number of patients in our study does not allow to draw definite conclusions from this finding. However, we assume that for severely affected patients with 10 or less points the intervention period was too short to achieve a substantial improvement. Alternatively, these patients were too severely affected to improve at any time.

While the primary outcome measure did not reveal beneficial effects of the intervention, we observed changes at neurophysiological level in the secondary outcome measure. The measure of motor excitability showed a significant increase in MEP amplitudes on the affected side in the intervention but not in the control group. Moreover, increased MEP amplitudes were correlated with the improvement of motor functions and the amount of upper extremity-focused therapy. The findings indicate that motor function improvements were mirrored by an increase in motor excitability and that the number of therapies modulated motor excitability changes. However, although the intervention group developed a stronger increase of motor excitability the motor performance was not superior to the control group. We speculate that the stronger increase of excitability might have preceded a stronger improvement of motor function which might have occurred with a longer duration of the intervention, e.g. 6–8 weeks. The question how increased excitability translates to clinical improvements certainly needs further attention in future studies.

We did neither find a significant change of cSP duration nor a correlation between cSP duration changes and motor function changes. As frequently described before, cSP duration was longer on the affected side compared to the non-damaged side [47–50]. This prolongation tended to become less pronounced after the intervention period, thus a trend towards a normalization (shorter cSP) was observed. It is difficult to interpret this trend when taking the small number of subjects into account. In order to address this issue a larger group of patients needs to be examined.

No adverse events and discontinuations were documented in this study. For some patients, the additional treatment sessions were challenging but feasible. The use of this robot-assisted arm training device was safe and acceptable for all participants included in the study.

## Conclusions

At behavioral level there was no additional benefit from the intensified robot-assisted therapy. However, the neurophysiological data showed a change associated with the degree of clinical improvement and the amount of overall upper extremity therapy. Motor excitability increases were more pronounced in the intervention group. However, it remains speculative whether the observed excitability changes were more sensitive than the clinical test. Future studies with a longer treatment period (e.g. six to eight weeks) need to explore the relationship between increase in excitability and improvement of function. In addition, it should be examined whether a further increase of the treatment period is feasible for the patients and the entire rehabilitation inpatient setting. Intensified robot-assisted therapy of the upper extremity appears to be a safe therapy.

## Data Availability

The datasets used and/or analysed during the current study are available from the corresponding author on reasonable request.

## Abbreviations

cSP: Cortical silent period
FMA-UE: Fugl-Meyer assessment for upper extremity
MEP: Motor evoked potential
MoCA: Montreal Cognitive Assessment
ms: Millisecond
MT: Motor threshold
mV: Millivolt
MVC: Maximum voluntary contraction
n: Number
TMS: Transcranial magnetic stimulation
µV: Microvolt
